# Carbon footprint of Pars plana vitrectomies in rhegmatogenous retinal detachment in Germany between 2013 and 2023 - A cohort study of fluorinated gas emissions

**DOI:** 10.1007/s00417-025-06905-7

**Published:** 2025-07-16

**Authors:** Piotr Strzalkowski, Alicja Strzalkowska, Mathias Roth, Sema Kaya, Gerd Geerling, Rainer Guthoff

**Affiliations:** Department of Ophthalmology – Medical Faculty, University Hospital Düsseldorf – Heinrich-Heine-University Düsseldorf, Moorenstrasse 5, Düsseldorf, 40225 Germany

**Keywords:** Rhegmatogenous retinal detachment, Fluorinated gas, Pars plana vitrectomy, Carbon emission, Carbon footprint

## Abstract

**Purpose:**

Climate change, largely driven by greenhouse gas emissions, is a global threat. The healthcare sector contributes significantly − 4.4% of global and 5.2% of Germany’s CO_2_ emissions. Rhegmatogenous retinal detachment (RRD) is a common ophthalmic emergency requiring surgical intervention to prevent blindness. Fluorinated gases (FG) such as SF_6_, C_2_F_6_, and C_3_F_8_ are routinely used in pars plana vitrectomy (ppV) due to their expansive properties, but they have a strong greenhouse effect. With an European Union-wide ban under consideration, this study assesses the associated emissions from ppV for RRD in Germany.

**Methods:**

We analyzed DRG-coded ppV data for RRD (2013–2023) from the German Federal Statistical Office. A national survey of vitreoretinal surgeons provided information on endotamponade preferences. CO_2_-equivalent emissions (CO_2_EM) were calculated using GWP100 values from the IPCC report.

**Results:**

Between 2013 and 2023, 354,505 ppV for RDD were performed in Germany. Gas tamponade was used in 55.3% of cases, silicone oil in 27.7%, and air in 16.9%. FG resulted in 201.34 tons of CO_2_EM. SF_6_ contributed 170.10 tons (84.5%), C_2_F_6_ 26.84 tons (13.3%), and C_3_F_8_ 4.40 tons (2.2%). This represents 18.3 tons of CO_2_EM annually, corresponding to 0.000003% of Germany’s total CO_2_EM and 0.00003% of healthcare sector emissions.

**Conclusion:**

This first comprehensive analysis of FG consumption and CO_2_EM in ppV for RRD in Germany shows that FG contribute a negligible portion to Germany’s and healthcare-related CO_2_EM. Gas tamponade remains crucial for the treatment of RDD and should therefore not be banned until proven alternatives exist. Further studies are needed to explore alternatives.

## Key messages

*****What is already known***:**

- Fluorinated gases such as sulfur hexafluoride (SF₆), hexafluoroethane (C₂F₆), and octafluoropropane (C₃F₈) are commonly used in pars plana vitrectomy (ppV) for rhegmatogenous retinal detachment (RRD) and have a high global warming potential, leading to planned restrictions in the European Union.

*****What this study adds***:**

- This is the first study to quantify the carbon footprint of ppV for RRD in Germany, analyzing 196,177 gas tamponade procedures performed between 2013 and 2023.
- These procedures resulted in 201.34 tons of CO₂ equivalent emissions, averaging 18.3 tons annually. SF₆ alone contributed 170.10 tons (84.5%), C₂F₆ 26.84 tons (13.3%), and C₃F₈ 4.40 tons (2.2%).
- Annual emissions from these procedures represent only 0.00003% of the German healthcare sector’s emissions (26.22 million tons) and 0.000003% of Germany’s total emissions (596.15 million tons).
- A European Union-wide ban on these gases poses a significant risk to patient care for sight-threatening RRD in the absence of medically equivalent alternatives.

## Background

Climate change is a main threat to our planet and human health in the 21 st century, driven by rising greenhouse gas emissions, deforestation, waste production and population growth [1, 2]. In 2022, the United States alone emitted 6.343 million tons of carbon dioxide (CO_2_) equivalent emissions (CO_2_EM), with CO₂ comprising 79.9% of these emissions [3]. This worldwide increase in greenhouse gases [4, 5] has fueled potentially irreversible global warming, leading to rising temperatures, sea levels, extreme weather events, and emerging diseases. To counter these effects and limit global warming to below 2 °C [6], all industries, including healthcare [7, 8], must reduce emissions.

In 2019, the global healthcare sector had a climate footprint of 2.0 gigatons CO_2_ emissions which are 4.4% of global CO_2_ emissions, highlighting its significant contribution to global greenhouse gas emissions [9]. This impact is more pronounced in specific countries, with the healthcare sector accounting for 8–10% of emissions in the United States, 25% of public sector emissions in the United Kingdom (NHS), and 5.2% of total emissions in Germany [9]. In Germany, greenhouse gas emissions from the healthcare sector increased from 63 million tons in 2014 to 66 million tons in 2015 and reached 68 million tons of CO_2_EM in 2019 [10]. This upward trend highlights the urgent need for targeted strategies - such as achieving net-zero emissions in medicine [11–14] - to reduce the sector’s environmental impact.

Rhegmatogenous retinal detachment (RRD) is a severely vision threatening disease [15] with increasing incidence [16–18] treated frequently by pars-plana vitrectomy (ppV) [19]. Per- and polyfluorinated alkyl substances (PFAS: sulfur hexafluoride (SF_6_), hexafluoroethane (C_2_F_6_) and perfluoropropane (C_3_F_8_)) are successfully used as expansive gas-air mixtures as endotamponade in ppV especially for RRD since 1973 [20].

However, SF_6_ gas was classified as one of the six greenhouse gases in the Kyoto Protocol in 1997 by the Parties to the United Nations Framework Convention on Climate Change (UNFCCC) [21]. While the proportion of fluorinated gases including SF6 is low (3.1%) compared to CO2 (79.7%), the global warming potential (GWP) of SF6 is much higher (23,900) than that than of CO_2_ (1) [22, 23]. The 125th German Medical Assembly in 2021 called for the German healthcare system to achieve climate neutrality by 2030 (Resolution II-03) and urged all decision-makers to “pursue this goal in a determined, consistent and timely manner“ [24]. A proposed European Union-wide ban on PFAS gases starting in 2023 has sparked significant discussion, as their use currently seems unavoidable in everyday ophthalmology practice.

In addition to having a high global warming potential, PFAS are highly persistent chemicals that break down very slowly due to their strong carbon-fluorine bonds and remain in the environment for thousands of years [25, 26]. They accumulate in ecosystems, posing long-term risks to wildlife and human health [27]. Examples of common PFAS include perfluorooctanoic acid (PFOA), which is used in non-stick cookware and water-repellent fabrics [28] and perfluorooctane sulfonate (PFOS), which was formerly used in firefighting foams and stain repellents [29].

Highly purified silicone oils or filtered air can serve as PFAS-free tamponades, but their indications differ from those of gas endotamponades. In addition, silicone oil tamponade related complications such as secondary glaucoma [30], cataract [31] and keratopathy [32] may occur. Furthermore, the risk of silicone retinotoxicity [33], associated with central vision loss, is discussed [34, 35], as well as reduced choroidal thickness [36], and the rare migration of silicone oil into the subretinal or intracranial areas [37]. Conversely, the routine use of silicone oil would require a mandatory second procedure for removal, which not only increases costs but also exposes patients to additional surgical risks and recovery time [38, 39]. The use of pure air for tamponade is associated with poorer outcomes in RDD treatment, with limited data overall [40].

Adopting climate-conscious practices in the healthcare sector is crucial for reducing greenhouse gas emissions and protecting both the environment and public health. Therefore, the aim of our study is to estimate for the first time the carbon footprint of fluorinated gases in Germany, which are used as an essential component for successful ppV for the treatment of sight-threatening RRD [41].

## Methods

In our retrospective study, we queried the German Federal Statistical Office and analyzed DRG-coded data from 2013 to 2023. Data from the previous year is published in October. OPS codes (5-158.11, 5.158.12, 5-158.13, 5-158.41, 5-158.42, 5-158.43) distinguish between expansive gas, silicone oil, BSS, or pure air used for endotamponade, but not individual gases. A recent nationwide survey of vitreoretinal surgeons across German hospitals found that SF_6_ was used in 66%, C_2_F_6_ in 27%, and C_3_F_8_ in 6% of cases [41]. These values were applied to estimate the proportion of gas endotamponades used. The study followed the Strengthening the Reporting of Observational Studies in Epidemiology (STROBE) reporting guideline [42].

### CO_2_EM calculation

The calculation of gas volume per surgical case was based on the use of one complete and sterile 40 mL canister per procedure, reflecting standard practice in our setting. The masses of SF_6_, C_2_F_6_, and C_3_F_8_ in canisters with non-expandable concentrations of 20%, 16%, and 12%, respectively, were determined by converting gas volume (mL) to mass (g) under standard temperature and pressure (STP) conditions. The resulting masses were 0.0521 g for SF6, 0.0394 g for C_2_F_6_, and 0.0403 g for C_3_F_8_. The CO_2_ equivalent mass (CO_2_EM) was determined by multiplying these values by their respective global warming potential (GWP100) from the IPCC6 (2021) report: SF6: 25,200, C_2_F_6_: 12,400, and C_3_F_8_: 9,290 [22] (Table 1). We have based these calculations on the calculator recently published by Moussa et al. [43]

**Table 1:** Global Warming Potentials (GWP) of Fluorinated Gases

Gas	Life time (years)	Global Warming Potential (GWP)		
		20 years	100 years	500 years
CO2	100–300	1	1	1
SF6	3200	18,300	25,200	34,100
C2F6	10,000	8940	12,400	17,500
C3F8	2600	6770	9290	12,400

Global Warming Potentials (GWP) for different time horizons. Table adapted from the Sixth Assessment Report of the Intergovernmental Panel on Climate Change (IPCC) [22]

All greenhouse gas emission values are expressed in metric tons of CO_2_EM, with one metric ton corresponding to 1,000 kg. This unit is used in accordance with international scientific and environmental standards (SI units).

To contextualize und to illustrate the CO_2_EM values for fluorinated gases used in ppV in Germany, we converted the values using the US EPA’s Greenhouse Gases (GHG) Equivalency Calculator [44], which estimates in gallons of gasoline and miles driven. These were then converted to liters (1 gallon = 3.78541 L) and kilometers (1 mile = 1.60934 km). Since the calculator’s conversion factors are not fully disclosed and may vary regionally, we applied data from the German Federal Environment Agency (2023), which estimates average fuel consumption at 7.7 L per 100 km, to convert gasoline equivalent into kilometers driven [45].

### Statistical analysis

Statistical analysis was performed using GraphPad Prism10 (GraphPad Software, San Diego, USA) for Mac. For statistical analysis, Categorical variables were presented as absolute and relative frequencies, whereas mean and standard deviation were computed for approximately normal-distributed continuous variables, otherwise median and interquartile range. Evaluation of data normality was performed using the Shapiro-Wilk test. Fisher’s Exact Test was used to evaluate the association between categorical variables. Linear regression analysis was employed to model the relationship between the number of retinal detachment cases and the year. All statistical tests were two-sided and *p*-value < 0.05 was considered statistically significant.

## Results

Between 2013 and 2023, a total of 354,505 cases of ppV for RRD were reported in Germany. The annual number of RDD cases increased continuously from 25,615 in 2013 to 38,880 in 2023, an overall increase of 51.7% (*p* < 0.0001). From 2013 to 2023, the incidence of RRD in Germany increased from 31.7 to 45.9 per 100,000 population (*p* = 0.0003). The average annual increase in total RDD cases during the study period was 1,207 cases per year, with the highest between 2022 and 2023 at 2,307 cases (Table 2).

**Table 2:** Pars Plana Vitrectomy Cases, Percentages, and Incidence for Retinal Detachment in Germany (2013–2023)

Years	Air	Gas	SO	Total	Incidence per 100,000
2013	4306	13,450	7859	25,615	31,7
2014	4915	14,731	7995	27,641	34,0
2015	5007	16,079	8229	29,315	35,7
2016	5131	16,766	8355	30,252	36,7
2017	5300	17,328	8705	31,333	37,8
2018	5582	18,915	9316	33,813	40,7
2019	5721	18,841	9328	33,890	40,7
2020	5577	18,230	8954	32,761	39,4
2021	6114	19,353	8965	34,432	41,4
2022	6146	20,606	9821	36,573	43,4
2023	6172	21,878	10,830	38,880	45,9
n	59,971	196,177	98,357	354,505	
%	16,9	55,3	27,7	100,0	

Total cases, percentages of pars plana vitrectomies and incidence for rhegmatogenous retinal detachment in Germany between 2013 and 2023 based on air, gas or silicone oil endotamponade

**Fig. 1:**
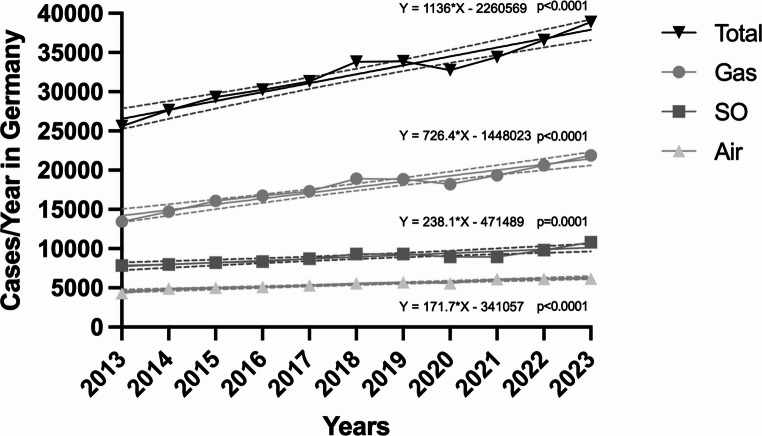
Total Pars Plana Vitrectomies for Retinal Detachment in Germany (2013–2023) by Endotamponade Type. Total cases of pars plana vitrectomies for rhegmatogenous retinal detachment in Germany between 2013 and 2023 based on air, gas or silicone oil endotamponade

Gas was the most frequently used endotamponade, accounting for 55.3% (196,177 cases) of all ppV during the observation period. The annual number of cases involving gas increased steadily from 13,450 in 2013 to 21,878 in 2023, representing a total increase of 62.7% (*p* < 0.0001). The mean annual increase in gas usage was 843 cases per year (Fig. 1).

Silicone oil (SO) tamponade was used in 27.7% (98,357 cases) of ppV. Annual case numbers for SO increased from 7,859 in 2013 to 10,830 in 2023, corresponding to a total increase of 37.8% (*p* = 0.0001). The mean annual increase in SO usage was 271 cases per year (Fig. 1).

Air endotamponade was used in 16.9% (59,971 cases) of ppV, showing the least variability over time. The annual number of cases with air endotamponade increased slightly from 4,306 in 2013 to 6,172 in 2023 (*p* < 0.001). This represents an overall increase of 43.3% over the study period, with an average annual change of 170 cases per year (Fig. 1).

The proportional distribution of endotamponade types remained stable throughout the observation period. Gas tamponade consistently accounted for the majority of RDD surgeries each year, followed by SO and air. Cumulatively, gas tamponade showed the largest absolute and relative increase in use, contributing significantly to the overall increase in vitrectomies, while the use of SO also increased steadily. In contrast, air tamponade showed a moderate upward trend, with a slight deceleration in growth in recent years.

## CO_2_ emission for fluorinated gas endotamponades in Germany

The total of 196,177 ppV for RDD with fluorinated gas endotamponades were performed in Germany between 2013 and 2023 correspond to 201.34 tons of CO_2_EM. With 170.10 tons of CO_2_EM (84.5% of total CO_2_EM), SF_6_ was the largest contributor among the gases used. C_2_F_6_ contributed 26.84 tons (13.3%), while C_3_F_8_ was responsible for 4.4 tons (2.2%).

This corresponds to CO_2_EM of approximately 18.3 tons per year, of which 15.5 tons come from SF6, 2.4 tons from C_2_F_6_ and 0.4 tons from C_3_F_8_ and corresponds to approximately 0.000003% of total German CO_2_ emissions from all sectors in 2019, which amounted to 596.15 million tons. SF_6_ accounts for 15.46 tons, or about 0.000003% of the total. C_2_F_6_ contributes 2.44 tons, or about 0.0000004%, and C_3_F_8_ 0.40 tons, or 0.00000007% (Table 3).

**Table 3:** Total CO_2_ Emissions from ppV with Fluorinated Gases and Share of Germany’s National Emissions

Gas	Total Emissions (CO_2_EM in tons)	% of Total CO_2_EM	Annual Emissions (CO_2_EM/year in tons)	% of Germany’s Total CO_2_EM (596.15 M tons in 2023[5])
Total (All Gases)	201.34	100	18.30	0.000003
SF_6_	170.10	84.5	15.46	0.000003
C_2_F_6_	26.84	13.3	2.44	0.0000004
C_3_F_8_	4.4	2.2	0.40	0.00000007

CO_2_-equivalent emissions (CO_2_EM) from 196,177 pars plana vitrectomies with fluorinated gas endotamponades in Germany (2013–2023), including total, annual values, and their share of Germany’s CO_2_ emissions in 2019

Over the study period, total CO_2_EM correspond to the fuel consumption and driving distance of 47 petrol-powered vehicles. Annually, this equates to approximately 60,031 km driven using 7,796 L of fuel. SF₆ accounted for 40 vehicles (50,709 km/year, 6,578 L/year), C_2_F_6_ for six (8,002 km/year, 1,038 L/year), and C_3_F_8_ for one (1,312 km/year, 170 L/year). Detailed values are provided in Table 4.

**Table 4:** CO_2_ Emissions from ppV with Fluorinated Gases converted to Fuel Use and Vehicle Equivalents in Germany

Gas	Equivalent Vehicles	Fuel Consumption (liters)	Distance Driven (km)	Fuel Consumption (liters/year)	Distance Driven (km/year)	Avg. Distance per Vehicle (km/year)
Total (All Gases)	47	85,758	660,336	7,796	60,031	14,06
SF_6_	40	72,453	557,888	6,578	50,709	13,947
C_2_F_6_	6	11,432	88,026	1,038	8,002	14,671
C_3_F_8_	1	1,873	14,422	170	1,312	14,429

CO_2_-equivalent emissions from fluorinated gas use in pars plana vitrectomies in Germany (2013–2023), expressed as fuel consumption and vehicle equivalents, including total and annual values

The environmental impact of fluorinated gases is demonstrated by their CO_2_EM per gram. Throughout the study period, SF_6_ emitted 1.31 kg CO_2_EM per gram, C_2_F_6_ 0.49 kg/g and C_3_F_8_ 0.37 kg/g. Based on their mass concentrations per unit volume − 0.0521 g for SF_6_, 0.0394 g for C_2_F_6_ and 0.0403 g for C_3_F_8_- the resulting CO_2_EM are 1.3137 kg, 0.4886 kg and 0.3741 kg, respectively. These values highlight the significant global warming potential of these gases, with SF₆ having the highest environmental impact. To put these results in context, the total CO_2_EM from ppV for RRD in 2023 represents only 0.000034% of Germany’s total CO_2_ emissions in 2023, which amounts to 596.15 million tons [5].

If all 129,477 cases currently operated in Germany with SF_6_ were replaced with C_2_F_6_, the CO₂EM could be reduced from 170.1 tons to 63.3 tons (−37%). This would result in a potential saving of 106.8 tons of CO_2_EM.

## Discussion

To the best of our knowledge, this study is the first comprehensive analysis of the use of fluorinated gases and carbon dioxide (CO_2_) emissions (CO_2_EM) in pars plana vitrectomy (ppV) for rhegmatogenous retinal detachment (RRD) in Germany. For effective retinal tamponade, the key properties of intraocular gases - surface tension and buoyancy - are essential. The surface tension maintains the cohesion of the bubble, allowing it to seal the retinal breaks and prevent the entry of fluid into the subretinal space. The buoyancy, which is proportional to the gas volume, provides an upward force to position the bubble over the retinal tear [46]. The primary role of the gas is to keep aqueous fluid away from the break until a chorioretinal adhesion forms after vitrectomy and subretinal fluid removal [47].

Fluorinated gases, including SF_6_, C_2_F_6_ and C_3_F_8_, are commonly used in ppV as tamponades to aid in the reattachment of the retina. Although used in small quantities, these gases have extremely high global warming potentials - often thousands of times greater than CO_2_ - making them far more effective at trapping heat [22]. SF_6_ has for instance much longer atmospheric lifetimes compared to CO_2_, with 1 kg of SF_6_ having a GWP of 23,900 kg of CO_2_ over 100 years [22, 23]. Moreover, different types of gas cylinders used in vitreoretinal surgery may contain enough gas for up to 12,000 procedures, but they typically expire after three years. Hospitals usually keep more than one cylinder on hand. As a result, it is possible that approximately 90% of the gas could go unused and be wasted [23].

Between 2013 and 2023, a total of 354,505 PPV cases for RRD were reported in Germany. During this period, the use of gas endotamponade increased steadily from 7,307 cases in 2013 to 12,339 in 2023, totaling 196,177 procedures - representing 55.3% of all ppV performed. The rising use of fluorinated gases in RRD treatment underscores the need to consider their environmental impact. Given their high global warming potential and long atmospheric lifespan, their use in vitreoretinal surgery should be evaluated in the context of sustainable healthcare.

Total emissions from all gases in our study amount to 18.3 tons per year, corresponding to approximately 0.000003% of Germany’s total CO_2_EM of 596.15 million tons in 2023. This represents about 0.00003% of the healthcare sector’s 68 million tons of CO_2_EM in 2019. SF_6_ accounts for 0.00002%, C_2_F_6_ for 0.000004%, and C_3_F_8_ for 0.0000006% of emissions within the sector. These results show that emissions from these gases make up only a very small fraction of the German healthcare sector’s total CO_2_EM.

In comparison, the UK has reported fluorinated gas emissions within its healthcare sector: in 2019, the total carbon footprint of the NHS was 25 megatons of CO_2_ [48]. Fluorinated gases used in vitreoretinal surgery contributed approximately 0.0037% of this total. Depending on the size of the gas cylinders used, the CO_2_ footprint of these procedures could range from approximately 0.0002–0.0075% of the NHS’s annual emissions [23]. In clinical practice, many retinal surgeons prefer gas endotamponade (GT) over air endotamponade (AT), as AT is associated with less favorable outcomes in rhegmatogenous retinal detachment (RDD) cases [41]. Current evidence supporting the use of air tamponade (AT) remains limited and is largely confined to carefully selected cases of focal and less extensive rhegmatogenous retinal detachment. Tan et al. demonstrated a significantly lower rate of primary reattachment in detachments involving the inferior quadrants when treated with AT (69.6%) as compared to conventional gas tamponade (84.7%; *p* = 0.009) [40]. Some authors have even proposed AT usage as a strategy to reduce CO_2_EM [49]. Despite these recommendations, AT was surprisingly used in only 47 of 2524 (0.9%) RRD repairs in a national study in the UK [50]. In contrast, vitreoretinal surgeons in Germany used AT in 16.9% of cases. Since 2021, however, this number seems to have plateaued. Despite the rising evidence, retinal specialists seem to prefer fluorinated GT over AT.

Since SF_6_ gas is classified as one of the six Greenhouse Gases (GHG) subject to usage restrictions [10], C_2_F_6_ or silicone oil may be considered as an alternative. Our results suggest that switching RRD procedures from SF_6_ to C_2_F_6_ could have a moderate environmental benefit, with a GWP of 12,400. If all 129,477 cases currently performed in Germany using SF_6_ were instead treated with C_2_F_6_, CO₂EM could be reduced from 170.1 tons to 63.3 tons, a 37% reduction. This substitution would result in a potential saving of 106.8 tons of CO_2_EM.

However, C_2_F_6_ requires a longer absorption time, which may be disadvantageous for patients rehabilitation due to restrictions on driving or flying [51].

The routine implementation of silicone oil endotamponade is also unfavorable due to a heterogeneous range of indications, a mandatory second procedure and consequently higher healthcare costs, as well as silicone oil-specific complications such as visual deterioration, inflammation and secondary glaucoma [35, 38, 52, 53].

To better define the indications for air tamponade in retinal detachment surgery, especially to ensure patient safety and optimize postoperative outcomes, validated data from prospective studies are needed.

As a first step to reduce carbon footprint in RRD surgery, vitreoretinal surgeons could make a change in their treatment regimen and use C_2_F_6_ in place of SF6. This change would not only have a positive impact on the environment but could also be a contribution to the sustainability of the healthcare sector. By adopting C_2_F_6_ as the primary GT, a significant reduction in CO_2_ emissions could be achieved, supporting both clinical efficacy and environmental responsibility in ppV for RRD.

### Strengths and limitations

A limitation of the study is that the German OPS coding does not allow a precise breakdown of the gases used (SF_6_, C_2_F_6_, C_3_F_8_). Another limitation of this study is that our estimation shows the minimal usage of gases based on gas delivery systems used in our clinic because of lack of knowledge concerning those systems in another clinics. Data were obtained from the German Federal Statistical Office and are based on hospital records; thus, outpatient treatments of retinal detachment may not be included. However, most retinal detachments in Germany are treated in hospitals. The policy makers must enable and improve detailed coding concerning type of gases and gas delivery system accordingly. A strength of this study is that it incorporates gas usage data and derives its estimates from the total number of RDD surgeries performed across Germany, rather than relying on extrapolations from a limited number of centers. Additionally, in the absence of national-level data on the types of RDD surgeries being conducted, the authors based their estimates on responses from the recent German-wide retina.net survey regarding current practice in RRD surgery [41] which addressed these concerns.

## Conclusions

The carbon footprint from fluorinated gases used in pars plana vitrectomy (ppV) for rhegmatogenous retinal detachment (RRD) accounts for a negligeable proportion of both the healthcare sector’s CO_2_ emissions and Germany’s total CO_2_EM. The 18.3 tons of CO_2_EM generated annually by these gases represent only 0.00003% of the healthcare sector’s emissions and 0.000003% of national emissions. While efforts to reduce avoidable emissions are important given their significant global warming potential, a complete European Union-wide ban on per- and polyfluorinated alkyl substances (PFAS), which include fluorinated gases in ophthalmology, could severely compromise patient care. Currently, there are no equivalent medical alternatives for RRD surgery. Policy makers must ensure a balance between environmental objectives and clinical needs to avoid compromising patient health. Further research into sustainable alternatives, including lower GWP gases or alternative tamponades, is essential to minimize environmental impact while maintaining clinical efficacy.
